# Precipitation and Soil Moisture Spatio-Temporal Variability and Extremes over Vietnam (1981–2019): Understanding Their Links to Rice Yield

**DOI:** 10.3390/s22051906

**Published:** 2022-03-01

**Authors:** Luyen K. Bui, Joseph Awange, Dinh Toan Vu

**Affiliations:** 1 Faculty of Geomatics and Land Administration, Hanoi University of Mining and Geology, Hanoi 100000, Vietnam; buikhacluyen@humg.edu.vn (L.K.B.); dinhtoan.vu@get.omp.eu (D.T.V.); 2 Department of Civil and Environmental Engineering, University of Houston, Houston, TX 77386, USA; 3 School of Earth and Planetary Sciences, Spatial Sciences, Curtin University, P.O. Box U1987, Perth, WA 6845, Australia; 4 Géosciences Environnement Toulouse (GET), CNRS, CNES, IRD, Université de Toulouse, 31400 Toulouse, France

**Keywords:** climate change, climate extremes, CHIRPS, TRMM, GLDAS, MERRA, Vietnam, rice yield

## Abstract

Vietnam, one of the three leading rice producers globally, has recently seen an increased threat to its rice production emanating from climate extremes (floods and droughts). Understanding spatio-temporal variability in precipitation and soil moisture is essential for policy formulations to adapt and cope with the impacts of climate extremes on rice production in Vietnam. Adopting a higher-order statistical method of independent component analysis (ICA), this study explores the spatio-temporal variability in the Climate Hazards Group InfraRed Precipitation Station’s (CHIRPS) precipitation and the Global Land Data Assimilation System’s (GLDAS) soil moisture products. The results indicate an agreement between monthly CHIRPS precipitation and monthly GLDAS soil moisture with the wetter period over the southern and South Central Coast areas that is latter than that over the northern and North Central Coast areas. However, the spatial patterns of annual mean precipitation and soil moisture disagree, likely due to factors other than precipitation affecting the amount of moisture in the soil layers, e.g., temperature, irrigation, and drainage systems, which are inconsistent between areas. The CHIRPS Standardized Precipitation Index (SPI) is useful in capturing climate extremes, and the GLDAS Standardized Soil Moisture Index (SSI) is useful in identifying the influences of climate extremes on rice production in Vietnam. During the 2016–2018 period, there existed a reduction in the residual rice yield that was consistent with a decrease in soil moisture during the same time period.

## 1. Introduction

Vietnam is located in southeast Asia and is well-known for its rice production, where it lies amongst the top three rice producers globally [1]. Domestically, its rice sustains a livelihood of more than 96 million people. In 2018, for example, rice exports earned Vietnam $5.6 billion (i.e., 9% of the world’s total rice export), thereby signifying the importance rice plays in the Vietnamese economy [2,3]). However, rice production in Vietnam depends heavily on the water supply, which is affected by climate extremes, e.g., floods and droughts [4,5]. This is because rice yield is vulnerable to drought/flood stress due to its shallower rooting than other crops [6]. Therefore, the knowledge of precipitation and soil moisture variability is essential to inform policy and adaptation strategies in order to prepare for climate extremes to minimize impacts on Vietnamese rice productivity [1].

In Vietnam, climate change has a significant influence on the amount of precipitation [7]. This, together with aquifer overexploitation, results in the drawdown of groundwater and causes surface subsidence [8]. However, a thorough understanding of spatio-temporal patterns of precipitation and soil moisture is challenging due to the lack of the most recent data, on the one hand, and the limitation in the spatial-temporal resolution of the available data on the other hand. Studies conducted on spatio-temporal patterns of precipitation in Vietnam so far have either been based on (i) irregularly-spaced in situ data [9,10,11] and/or fairly old gridded data [12,13], or (ii) dedicated to local study areas, such as one or several agro-ecological regions or provinces [14,15,16,17]. Studies focusing on spatial-temporal patterns of precipitation over Vietnam have been investigated in the literature [18,19], but similar studies on soil moisture are non-existent. Furthermore, most of the existing studies based on remotely-sensed data are not the most recent and are dedicated to local areas rather than the whole of Vietnam [20,21] and do not present the association of these variabilities to rice production.

Because of the continuous impacts of climate extremes, a spatio-temporal variability assessment of updated precipitation and soil moisture data is vital for understanding their influence on rice production for Vietnam. For instance, Bhowmik and Costa [22] investigated the influence of the variability in precipitation on rice productions in Bangladesh. Zheng et al. [23] considered the impacts of variability in soil moisture on rice production in China, while Ahmad et al. [24] analyzed the influence of water productivity on a rice-wheat cropping system in Pakistan. Other studies that have been undertaken to assess the impacts of variability in precipitation and soil moisture on rice production globally include the works of Tao et al. [25] and Stuecker et al. [26]. For Vietnam, however, no study of remotely-sensed gridded precipitation and soil moisture data for their spatio-temporal variabilities and their associated impacts on rice production has been published. In addition, no comprehensive analysis to compare the consistency of remotely-sensed precipitation and soil moisture products over Vietnam has been reported.

The objectives of this study are, therefore, three-fold: (i) to assess the spatio-temporal variability (in short-, medium-, and long-term) of selected remotely-sensed gridded precipitation and soil moisture products over Vietnam and their associated influence on rice yield for the 1981–2018 period, thereby informing the impacts of climate variability/change on rice yield; (ii) to identify and analyze the episodes of climate extremes (droughts); and (iii) to assess the impacts of climate extremes on the overall Vietnamese rice yield over the same time period. These investigations and assessments are implemented through the application of higher-order cumulant statistics of Independent Component Analysis (ICA) applied to the Standardized Precipitation Index (SPI), the Standardized Soil Moisture Index (SSI), and the soil moisture standardized anomalies. The novelty of the study is the assessment of whether or not Vietnamese rice yield has a link with climate indices derived from remote sensing-based precipitation or soil moisture products.

Due to the lack of in situ data, soil moisture from two products: the Global Land Data Assimilation System (GLDAS) and the Modern-Era Retrospective Analysis for Research and Applications Version 2 (MERRA-2), are cross-compared for their correlation prior to conducting the spatio-temporal analyses. These analyses will also reveal the impacts of climate change/variability in the short- and long-terms. Similarly, two precipitation products of Tropical Rainfall Measuring Mission (TRMM) and Climate Hazards Group Infra-Red Precipitation with Station data (CHIRPS) are investigated for their consistency over the Vietnamese mainland.

The remainder of the study is organized as follows. Section 2 introduces the study area, data adopted, and methods implemented in this study. In Section 3, the results are discussed, while Section 4 concludes the study.

## 2. Study Area, Data and Method

### 2.1. Study Area

**Figure 1:**
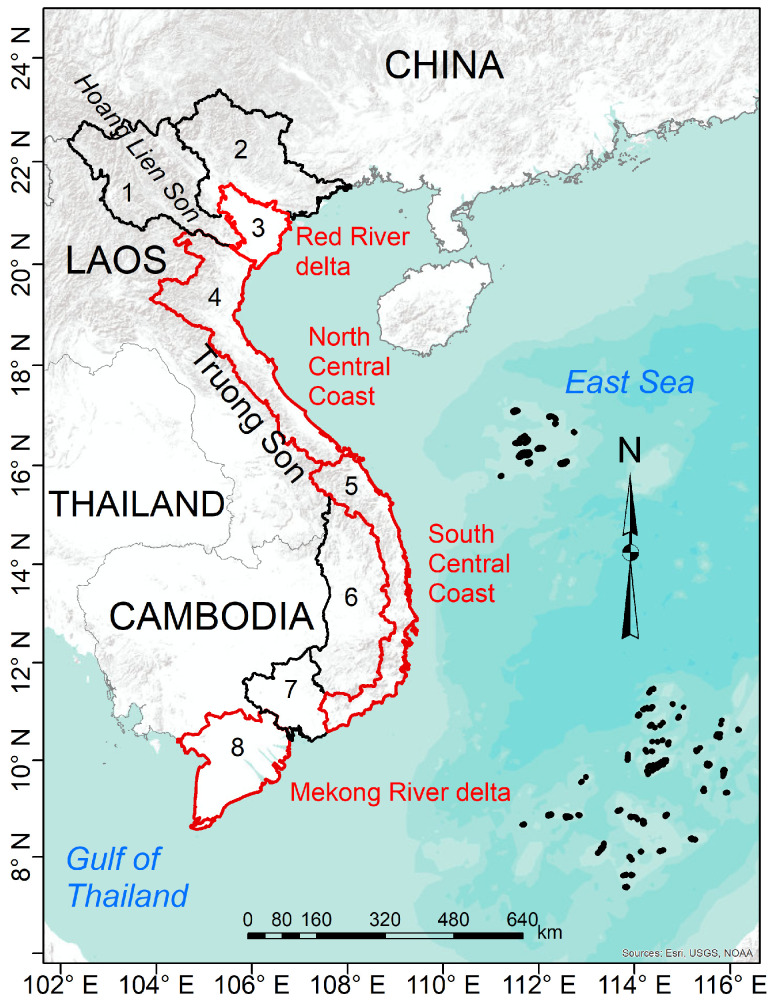
Vietnam map with eight agroecological areas: Northwest (1), Northeast (2), Red River Delta (3), Northern Central Coast (4), Southern Central Coast (5), Central Highlands (6), Southeast (7), Mekong River Delta (8). Red borders are associated with the main rice-producing areas.

Vietnam (Figure 1) is located in the easternmost part of the Indochina Peninsula, identified as one of the world’s richest and grandest natural places with substantial cultural diversity. It shares a border with China in the northern part, while the southernmost point is contiguous to the Gulf of Thailand. The longest borders lie in the west, where they are shared with Laos and Cambodia, and the East Sea lies to the east. Two mountainous areas are the Hoang Lien Son, which is located in the Northwest area, and the Truong Son, which stretches along the west border. There are two river delta regions in the Vietnamese mainland, the Red River Delta in the north and the Mekong River Delta in the south, that differ greatly in their hydrology, timing and extent of flooding, and their floral and faunal communities. This divergence resulted from climatic and geological differences and the nature of rivers that flow into them.

Vietnam’s mainland is divided into eight agro-ecological regions: Northwest (1), Northeast (2), the Red River Delta (RRD) (3), the North Central Coast (NCC) (4), the South Central Coast (SCC) (5), Central Highlands (6), Southeast (7), and the Mekong River Delta (MRD) (8). It is an agricultural country in which rice production is an important food supply and international export. Vietnamese rice is mainly produced in three main regions: the RRD (Region 3, ∼20%), the Central Coast area (Regions 4–5, ∼15%), and the MRD (Region 8, ∼50%) (see Figure 1). It should be noted that there is a slight difference in the agro-ecological region and climate sub-region classifications in that, in the latter approach, the Southeast (Region 7) and the MRD (Region 8) are merged into one sub-region named the Southern area [9,12,13,27]. In this study, the agro-ecological regions are adopted due to their direct association with Vietnamese rice yield statistics.

Due to its geographical shape, topography, and location in southeast Asia, Vietnam is closely associated with monsoons, including the southwest (summer) monsoon (May–October) and the northeast (winter) monsoon (November–April) [28]. In northern Vietnam, temperature and rainfall are known to be dominated by the seasonal cycle, with the hottest time being between June and August, while in the south, temperatures are less seasonal with wet and dry seasons [29]. Global warming has increased its effects worldwide, and Vietnam is no exception. The average temperature in Vietnam has increased at a rate of 0.26 °C every 10 years [28]. The Ministry of Natural Resources and Environment (MONRE) of Vietnam generated a report on climate change and sea level rise scenarios for Vietnam (version 2016, http://www.imh.ac.vn/files/doc/2017/CCS%20final.compressed.pdf, accessed on 1 December 2021). According to this report, by the end of the 21st century, the temperature in Vietnam is forecast to rise by 3.3–4.0 °C in the north and 3.0–3.5 °C in the south, while rainfall is predicted to increase with the highest predicted amount of over 20% for most areas.

### 2.2. Data

In this study, various products of precipitation, soil moisture, and temperature data were utilized, which will be described in the following sub-sections (Table 1).

#### 2.2.1. Vietnam Rice Statistics

**Figure 2:**
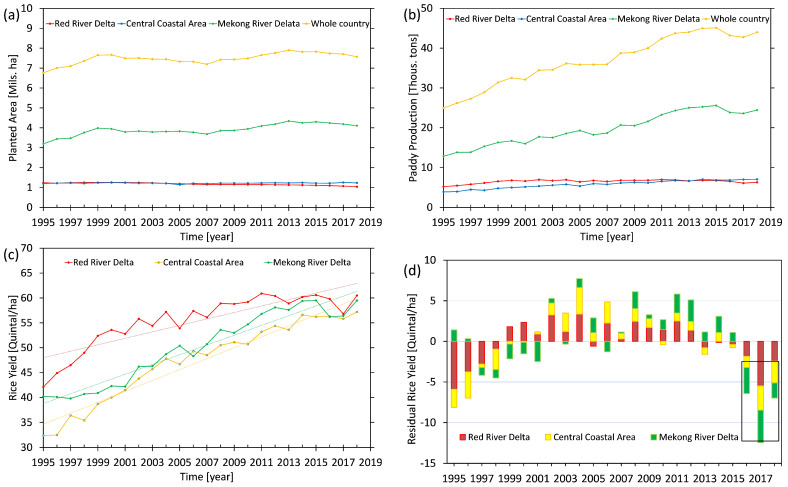
Statistics of planted area and rice over the whole of Vietnam and main rice-producing areas; (**a**) annually planted area, (**b**) rice production, (**c**) rice yield, and (**d**) residual rice yield after removing a linear trend. The rice yield was derived by dividing the rice production by the planted area.

The statistics of planted areas, rice production, and yield are gathered and provided by the General Statistics Office of Vietnam as sub-datasets included in the Agricultural, Forestry, and Fishing statistical dataset (https://www.gso.gov.vn/en/px-web/?pxid=E0614&theme=Agriculture%2C%20Forestry%20and%20Fishing, accessed on 12 September 2019). These statistics have been gathered from 1995. The annual rice yields (in quintal per hectare) were computed by dividing the rice productions (in thousands of tons) by planted areas (in millions of hectares). The demonstration of their changes is graphically shown in Figure 2 for the whole of Vietnam as well as the three main producing areas, including the RRD (Region 3, Figure 1), the Central Coast area (Regions 4 and 5, Figure 1), and the MRD (Region 8, Figure 1).

As can be seen from Figure 2, while the annual rice yield experiences an increasing trend as a result of, e.g., applications of new technologies, improving soil quality, use of improved rice seeds, extraordinary alterations can be seen in some periods. For example, reductions in rice production/yield can be seen in the years 2016–2018 (Figure 2b,c) due to drought impacts. Therefore, a linear trend was removed from the rice yield statistics (Figure 2c) to highlight the impact of climate extremes and dry soil moisture (Figure 2d).

#### 2.2.2. Precipitation

Two precipitation datasets, Tropical Rainfall Measuring Mission (TRMM) [31,32,33] and Climate Hazards Group Infra-Red Precipitation with Station data (CHIRPS) [30] were investigated in this study. The two datasets are slightly different in their spatial resolutions, which are $0.25^{\circ }\times 0.25^{\circ }$ (TRMM) and $0.05^{\circ }\times 0.05^{\circ }$ (CHIRPS), and the temporal coverage, i.e., 1998–present (TRMM) and 1981–present (CHIRPS). TRMM is a gridded rainfall dataset estimated from TRMM Multisatellite Precipitation Analysis (TMPA), published by the National Aeronautics and Space Administration (NASA) Goddard Space Flight Center (GSFC) [31,32,33]. The data has different temporal resolutions, such as monthly, daily, or sub-daily (3-hour), of which the former, validated in the Asian region, e.g., by Khandu et al. [39], is applied in this study. Its spatial coverage lies between [$50^{\circ }$ S, $50^{\circ }$ N] and [$180^{\circ }$ W, $180^{\circ }$ E]. In the present paper, TRMM-3B43 version 7 level 3 obtained from https://pmm.nasa.gov/data-access/downloads/trmm (accessed on 3 September 2019) was applied.

CHIRPS was principally developed for agricultural drought research [30]. It is provided at a similar spatial coverage to that of TRMM (i.e., between [$50^{\circ }$ S, $50^{\circ }$ N] and [$180^{\circ }$ W, $180^{\circ }$ E]). CHIRPS also comes with various temporal resolutions, e.g., daily, pentadal, and monthly. In this study, monthly CHIRPS version 2.0 obtained from https://data.chc.ucsb.edu/products/CHIRPS-2.0/ (accessed on 4 September 2019), was investigated. CHIRPS and TRMM are provided at millimeters per month (mm/month) and millimeters per hour (mm/hr), respectively. Therefore, in order to get a unified unit, TRMM was converted to mm/month by multiplying the hourly precipitation rate (i.e., mm/hr) with the total hours in the corresponding month. This was carried out on a pixel-by-pixel basis.

The two datasets were tested for their consistency over the entire Vietnam region (Appendix A). A high correlation with the maximum and minimum correlation coefficients being ∼0.97 and ∼0.53 at a 95% confidence level was found; a lower correlation was found in the SCC and Central Highland areas. The areal means of the two products over the four main rice-producing areas of the RRD, the NCC, the SCC, and the MRD indicate a high consistency with the smallest coefficient of 0.86 found in the SCC area and a value of ∼0.95 at a 95% confidence level derived in the remaining areas.

#### 2.2.3. Soil Moisture

In this study, two products of soil moisture, the Global Land Data Assimilation System (GLDAS) and the Modern-Era Retrospective Analysis for Research and Applications Version 2 (MERRA-2), were investigated for their link with the Vietnamese rice yield statistics. The reason for adopting both products is due to the lack of in situ soil moisture products; hence, the eventual deduction was based on the consistency of the results. GLDAS is jointly developed by the National Aeronautics and Space Administration (NASA), Goddard Space Flight Center (GSFC), the National Oceanic and Atmospheric Administration (NOAA), and National Centers for Environmental (NCEP) by using ground- and space-based observations [36]. GLDAS currently involves four land surface models, i.e., Mosaic [40], Noah [34,35], the Community Land Model (CLM) [41], and the Variable Infiltration Capacity (VIC) [42]. The widely-used model of Noah version 2.1 obtained from https://ldas.gsfc.nasa.gov/index.php/data (accessed on 2 September 2019) was adopted in this work. It is provided at a monthly temporal resolution covering the period between January 2000 and July 2019. There are two spatial resolutions provided, i.e., $1^{\circ }\times 1^{\circ }$ and $0.25^{\circ }\times 0.25^{\circ }$, of which the latter one was utilized in the present study. The spatial coverage is limited between [$60^{\circ }$ S, $60^{\circ }$ N] and [$180^{\circ }$ W, $180^{\circ }$ E]. The dataset is provided in the forms of soil layers at different depths (0–10 cm, 10–40 cm, 40–100 cm, and 100–200 cm) as well as the root zone layer (up to 100 cm). GLDAS soil moisture has been validated in several studies in the literature against in situ data or SPI time series, e.g., in the Tibetan Plateau [43], South America [44], and Austria [45], which indicated good agreement in terms of correlation coefficients.

The Modern-Era Retrospective Analysis for Research and Applications (MERRA) is a global reanalysis product generated based on the Goddard Earth Observing System version 5.2.0 (GEOS-5) atmospheric model and Data Assimilation System (DAS) published by NASA [38]. MERRA was developed on data of different types, such as wind, temperature, humidity, precipitation, moisture, and pressure, derived from various sources. MERRA version 2 (MERRA-2) [37] was introduced as a replacement of the original MERRA thanks to advances in the assimilation system, as well as new observations. MERRA-2 provides different datasets beginning from 1980 to the present in both the mainland and ocean, including soil moisture, snow/ice, and canopy water, together with other climatic data of different types. The dataset is provided with soil layers at different depths, including root zone (0–100 cm), surface (0–5 cm), and profile (from the surface down to the bedrock). MERRA-2 soil moisture has been validated against in situ measurements in, e.g., North America, Europe, and Australia [46], or globally [47], which indicated good agreement. Different from MERRA’s spatial resolution that is provided at a $0.667^{\circ }\times 0.5^{\circ }$ grid in longitude and latitude, MERRA-2 products are improved in the longitude spacing to $0.625^{\circ }$, while that of latitude is maintained. MERRA-2 comprises instantaneous and time-averaged productions of which the former are provided at synoptic or mid-synoptic times, while the latter is provided at hourly, three-hourly, monthly, or monthly diurnal temporal resolutions. Here, the monthly gridded soil moisture was utilized, which can be accessed at https://gmao.gsfc.nasa.gov/reanalysis/MERRA-2/data_access/ (accessed on 10 November 2019).

The two datasets at the equivalent root zone depth were tested for consistency (Appendix B). Due to the difference in spatial resolution, pixel-by-pixel consistency could not be tested. Instead, the areal mean time series of soil moisture changes over the four main rice-producing areas was tested, with the results generally showing higher correlations in the MRD (0.92) than in the other areas of RRD (0.57), NCC (0.39), and SCC (0.51). Due to the connection between precipitation and soil moisture, both of which may influence rice yield, GLDAS soil moisture and TRMM precipitation were tested for their correlation (Appendix C). GLDAS and TRMM were selected for this test because of their equivalence in spatial resolution (Table 1). The results showed that a lower correlation was exhibited in mountainous areas (i.e., the northwest and central Highlands) and coastal areas (i.e., the NCC and the SCC). The correlation coefficients varied between ∼0.34 and ∼0.85. The areal mean time series over the four main rice-producing areas showed correlation coefficients varying between 0.69 and 0.80, in which the deltas (RRD, MRD) exhibited higher correlations than the coastal areas (NCC, SCC)

Hereinafter, CHIRPS, with higher spatial resolution and longer time coverage (Table 1), was adopted as a representative of the remotely-sensed gridded precipitation data and applied to subsequent investigations. Similarly, with a higher spatial resolution, GLDAS was adopted as a representative of the remotely-sensed soil moisture product, though it is of shorter time coverage (Table 1). Additionally, with the emphasis on testing the influence of soil moisture on rice yield, the 10–40 cm depth sub-dataset was utilized, which matches best with rice roots with lengths up to ∼25 cm.

### 2.3. Methods

**Figure 3:**
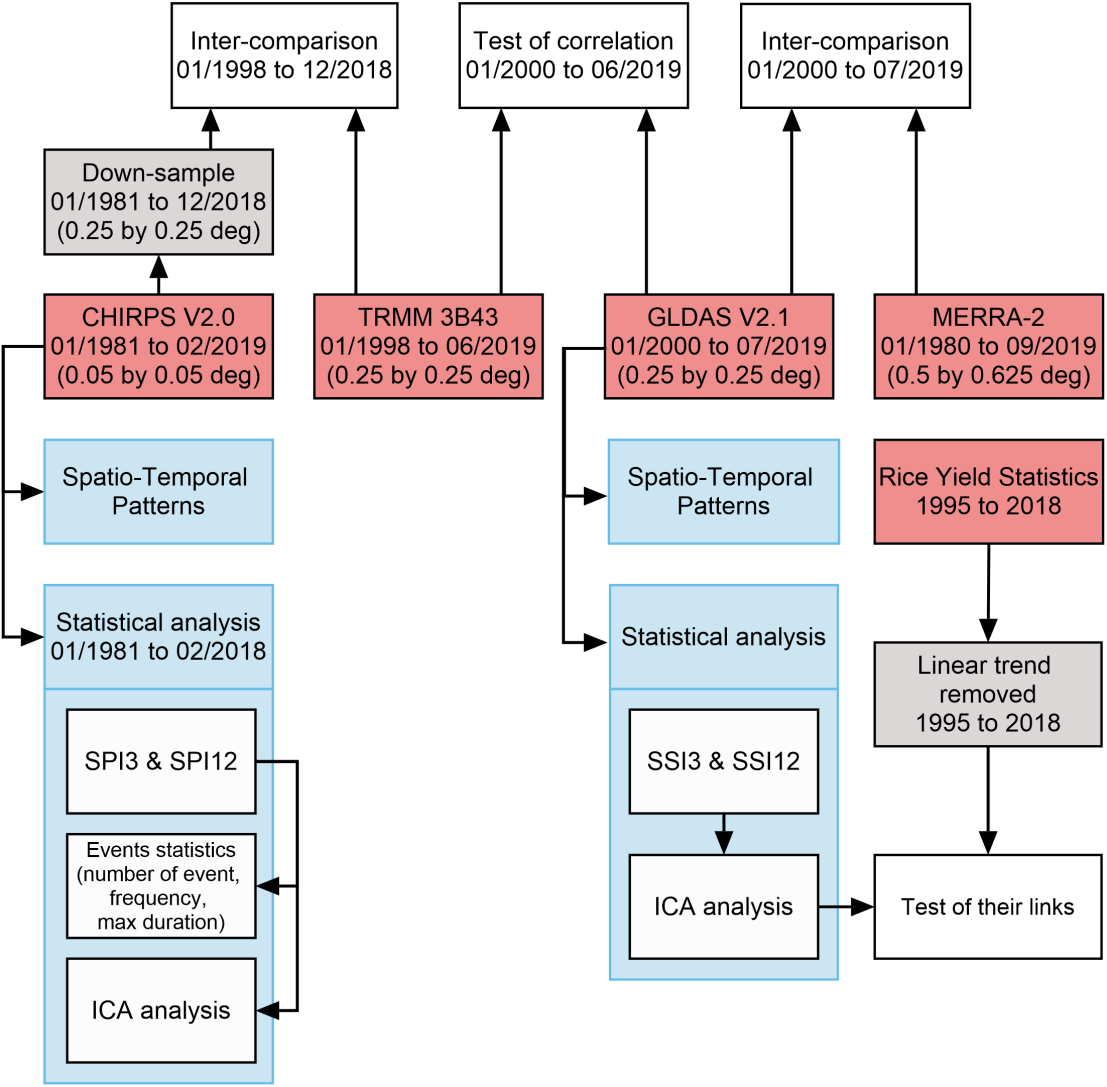
Graphical illustration of data and the processing steps employed in this study.

Figure 3 outlines the processing steps implemented in this study. The consistencies between CHIRPS and TRMM precipitation (Appendix A) and between GLDAS and MERRA-2 soil moisture (Appendix B) were tested first. Then, the correlation between TRMM precipitation (tested due to its equivalent spatial resolution to that of GLDAS) and GLDAS soil moisture was examined (Appendix C). The spatio-temporal patterns of precipitation and soil moisture were subsequently analyzed (Section 3.1). Drought conditions in Vietnam reflected by SPI were then investigated (Section 3.2). Finally, the impact of climate extremes on rice yield was assessed by SSI (Section 3.3). It is worth mentioning that different indices were tested in this study both to show the recent climate change effect in Vietnam, as well as its potential influence on Vietnamese rice yields. SPI is an index used to represent the amount and the change of precipitation measured at different areas and times; its drought classification standard (Table 2), therefore, shows drought significance. Additionally, with the aim to test the influence of climate variability/change on rice yield, SSI was utilized. SSI reflects soil moisture conditions, which are influenced by precipitation and irrigation. SSI is potentially affected by temperature and may reflect the missynchronization and lags with precipitation.

#### 2.3.1. Independent Component Analysis

Decomposition techniques are frequently adopted to reduce data dimensions as well as extract dominant signals in climatological data. Principal Component Analysis (PCA) [48,49,50] and Independent Component Analysis (ICA) [51,52,53,54] are among the mostly used approaches. The ICA can be considered as an extended version of the PCA [52,55]. The technique works based on the assumptions that the sources under separation are independent and the observations follow a non-Gaussian distribution. The ICA incorporating the Joint Approximate Diagonalization of Eigen-matrices (JADE) technique was applied in this study [51,56].

#### 2.3.2. Standardized Precipitation Indices

The SPI [57,58] is one of the indices most widely used in drought research due to its advantages (see, e.g., Awange et al. [59]). The SPI relies solely on precipitation and can be applied to various drought applications, e.g., in meteorology, hydrology, or agriculture. Moreover, in the SPI, various time scales can be adopted according to applications, e.g., 2–3-month time scales are appropriate for agricultural studies [60], 2–6-month should best describe stream flow characteristics [61], 5–24-month time scale are utilized for groundwater level research [62,63]. Furthermore, SPI is not affected by topography [64], but has disadvantages such as the dependence on the standardization of the index or the suitability of the theoretical probability distribution function that is chosen [62,65].

In SPI computation, monthly time series of precipitation is first aggregated at an appropriate time scale, e.g., 1-, 3-, 6-, 12-, 24-month, followed by the normalization where a chosen probability density function is fitted. Different probability functions have been tested in SPI analysis, e.g., gamma, Weibull, lognormal, or exponential functions [66,67]. The next step is to derive the cumulative distribution using a fitted function, before the standardized normal variates are derived. The commonly used workflow, incorporating the Maximum Likelihood Estimation (MLE) applied in fitting the gamma function, was applied in this work [68,69]. Once the SPI is computed, it can be used for drought/wetness classification by comparing with well-known thresholds, as shown in Table 2 (see Awange et al. [59] on the definition of drought).

The SPI at different time scales was adopted in this study. Firstly, with the focus on researching the influence of drought on agriculture (rice yield), the 3-month time scale SPI (hereinafter SPI3) was adopted. Secondly, the 12-month time scale SPI (hereinafter SPI12) was utilized as well, which reflects the hydrological drought. SPI12 was utilized due to that fact that, in Vietnam, rice is watered not only by rainfall but also by the irrigation system with water provided from rivers, streams, canals, and reservoirs.

#### 2.3.3. Standardized Anomalies

Different data tested in the present study are provided at non-identical spatial resolutions of which their magnitudes should be distinct. Therefore, a comparison of these data based directly on their values is impossible. Standardized anomalies were thus applied instead. Standardized anomalies have been widely applied to compare climatology data [60,70,71,72,73]. Various types of standardized anomalies can be adopted, including monthly standardized anomalies, where monthly means are removed before dividing by the monthly standard deviation; seasonal standardized anomalies, where a similar approach is implemented to mean values and standard deviation of data derived by aggregation over three consecutive seasonal months; or long-time standardized anomalies, where mean values and the standard deviation over the entire time period are used (data of at least 30 years are usually required to capture climatic change impacts).

These types are normally chosen based on an application. For instance, in agricultural applications, seasonal standardized anomalies are frequently applied due to the fact that three months are considered as a suitable time period for storing moisture in soil layers that are directly related to agricultural crops, and three months are normally the standard time period for agricultural production. However, in Vietnam, wet rice is produced all year with various crops, including Autumn crops (April–August), Winter crops (May/June–November/December), and Spring crops (November/December–April) [74]. The long-time standardized anomalies were thus applied in this study, derived by [70,71,72]:

(1) $$Z_{x,i,j}=\frac{X_{x,i,j}-\mu_{x}}{\sigma_{x}},$$

where, $Z_{x,i,j}$ is the standardized anomalies of pixel *x* in the *i*th month and *j*th year, $X_{x,i,j}$ is the corresponding monthly mean variable, $\mu_{x}$ and $\sigma_{x}$ are the long-term mean value and the corresponding standard deviation, respectively.

## 3. Results and Discussion

In this section, we investigate the spatio-temporal variability of the CHIRPS precipitation and GLDAS soil moisture for their monthly and annual means (Section 3.1). The 3-month and 12-month time scale CHIRPS SPI was calculated and statistically analyzed by the ICA. Statistics of SPI drought events, including the number of events, their frequencies, and maximum duration, were investigated thereafter (Section 3.2). Finally, the 3-month time scale SSI (SSI3) and 12-month time scale SSI (SSI12) were calculated, and the statistical analyses by ICA were tested for their link with rice yield (Section 3.3).

### 3.1. Spatio-Temporal Variability Analysis

#### 3.1.1. Spatio-Temporal Variability of Precipitation

Due to its geographical shape, topography, and location in southeast Asia, Vietnam is closely associated with monsoons that constitute two principal seasons: dry-cool Winter (November–March) and warm-wet Summer (April, May–September), separated by short transitional periods [29]. Precipitation has been widely documented to be dominated by seasonal patterns in northern Vietnam, while in the southern part, these characteristics are less dominant. The spatio-temporal variability of precipitation has been widely studied globally (e.g., [75]). In this sub-section, the spatio-temporal variability of CHIRPS precipitation in Vietnam is reported.

**Figure 4:**
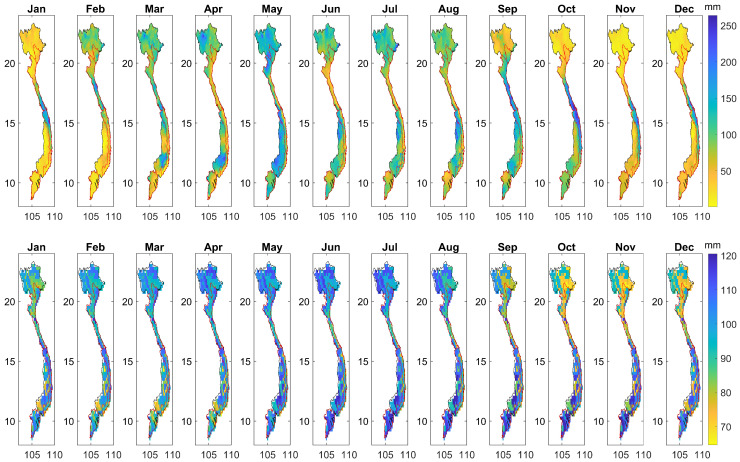
(**Top**) The monthly mean CHIRPS precipitation and (**bottom**) the monthly mean GLDAS soil moisture derived over the whole of Vietnam.

The monthly mean CHIRPS precipitation during the 1981–2019 period was calculated on a pixel-by-pixel basis and shown in Figure 4 (top). This confirms what has been widely documented by [29] that, in general, the rainy season lasts from April/May until September/October, which starts and ends earlier in the north (above 20${}^{\circ }$ N), around February and September, respectively. In addition, July–August receives the highest amount of precipitation over most parts of the country owing to the southwest monsoon [76]. In contrast, December to April experiences the driest time of the year, corresponding to the Winter season (December to February) and inter-monsoon (March to May) [77].

**Figure 5:**
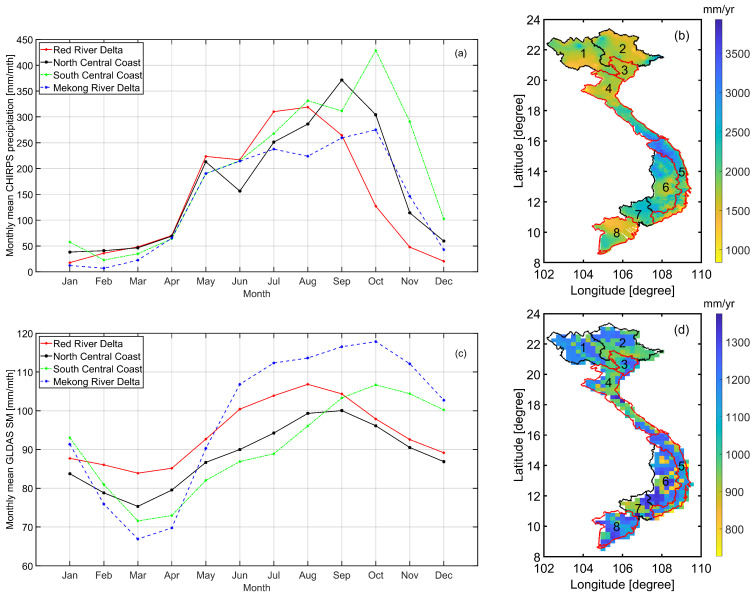
(**a**) Areal mean and (**b**) annual mean of monthly CHIRPS precipitation. (**c**) Areal mean and (**d**) annual mean of GLDAS monthly soil moisture. The areal means were computed over four main rice-producing areas and the annual means were computed for the whole of the Vietnamese mainland.

The spatially-averaged monthly precipitation over four areas of interest is depicted in Figure 5a where different peaks were found between areas. In the SCC and MRD areas, the peaks occurred in October, while August and September had the highest amount of precipitation in the RRD and NCC areas, respectively. In order to have a perspective of the spatial pattern of precipitation over Vietnam, the annual precipitation was computed by aggregating the monthly precipitation over each year for all pixels. As a result, the total annual precipitation of each year between 1981 and 2018 was derived. A 38-year mean annual precipitation was subsequently computed, of which the results are shown in Figure 5b. The results indicate that the annual mean precipitation varies within 841 mm/yr and 3930 mm/yr, with more precipitation received in the Central Coast, Central Highlands, and Southeast areas (Regions 4–7 in Figure 5b).

#### 3.1.2. Spatio-Temporal Variability of Soil Moisture

The spatio-temporal variability of soil moisture has been widely studied globally (e.g., [78]). In this sub-section, the spatio-temporal variability in GLDAS soil moisture was investigated. The monthly mean soil moisture was computed, of which, the results are depicted in Figure 4 (bottom). The results show that wetter periods, in terms of soil moisture, were different between areas, with a close association with the change of precipitation in time. Specifically, over the northern and NCC areas (above 15${}^{\circ }$ N), the wetter period is between February and August. In contrast, over the southern and SCC areas (below 15${}^{\circ }$ N), the wetter time starts latter in May. The monthly mean soil moisture was spatially averaged over the four main rice-producing areas, of which the results are shown in Figure 5c. There was agreement in the peaks of graphs, which reveals the months when the largest amount of precipitation was captured (Figure 5a), equivalent with those when the wettest soil moisture was detected (Figure 5c), which were August (RRD), September (NCC), and October (SCC and MRD).

The annual mean soil moisture was subsequently computed and is shown in Figure 5d. Disagreement in the spatial patterns between the annual mean of CHIRPS precipitation (Figure 5b) and the annual mean of GLDAS soil moisture (Figure 5d) was seen. We recall here that it is generally wetter in terms of precipitation in the Central Coast (Regions 4–5), Central Highlands (Region 6), and Southeast (Region 7) areas than the others, but this is not the case in terms of soil moisture, as shown in Figure 5d. This can be attributed to different factors that affect the amount of moisture in soil layers, including water provided by precipitation, the irrigation system, and the water loss caused by evapotranspiration and the drainage system. As a result, the change in GLDAS soil moisture and CHIRPS precipitation in time agreed (increased precipitation in time resulted in increased soil moisture), but the spatial patterns did not agree due to the inconsistency in other conditions between areas including temperature, irrigation, and drainage systems.

### 3.2. Drought Conditions Reflected by Spatio-Temporal Patterns of SPI

Here, the drought events identified by CHIRPS precipitation variability over the 1981–2018 time period were studied. To this end, SPI3 and SPI12 were investigated for their spatio-temporal patterns. The ICA analysis was adopted to decompose the dominant variability over the entire study area. The SPI3 and SPI12 were first computed for each pixel with the drought events defined as those shown in Table 2, following those of, e.g., Awange et al. [70]. A more elaborate definition of drought was presented in Awange et al. [59], which is slightly different from that adopted in this study. The number of drought events, their frequencies, and maximum duration during the study period of 1981–2018 were then calculated. The number of drought events was computed by counting the number of months with drought indices smaller than −1. The duration of drought events was defined as the length of consecutive months during which the drought indices were smaller than −1. Finally, the frequency of drought was estimated as the ratio of the number of drought months over the entire study length in percentage.

**Figure 6:**
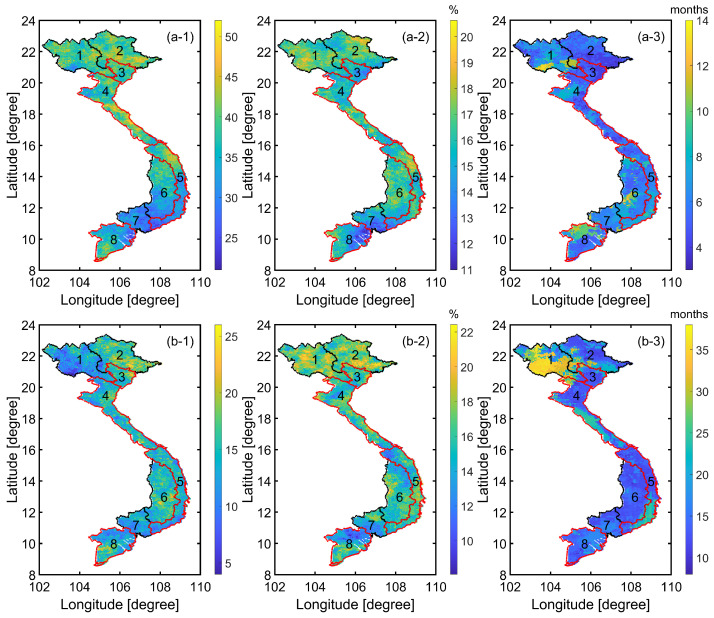
Characteristics of (**a**) SPI3 and (**b**) SPI12 drought events (defined by SPI <−1.0) during the study period of 1981–2018: (**a-1**,**b-1**) number of events, (**a-2**,**b-2**) frequency, (**a-3**,**b-3**) maximum duration. The sub-area borders and their order numbers are the same as those in Figure 1.

The computed results are depicted in Figure 6, in which an agreement exists in the spatial patterns between the number of events (Figure 6a-1,b-1) and their frequencies (Figure 6a-2,b-2). Within the 38-year period from 1981 to 2018, the maximum and minimum number of events were 52 (SPI3) and 26 (SPI12), and 21 (SPI3) and 4 (SPI12). The maximum frequencies were ∼20% (SPI3) and ∼22% (SPI12), and the minimum frequencies were ∼10% (SPI3) and ∼8% (SPI12). Generally, fewer events and lower frequencies were found in the lower half of the SCC (Region 5), the Central Highlands (Region 6), the Southeast area (Region 7), and the upper part of the Vietnamese MRD (Region 8). The maximum and minimum duration of drought events detected is 14 months (SPI3) and 38 months (SPI12), and 3 months (SPI3) and 8 months (SPI12) (see Figure 6a-3).

**Figure 7:**
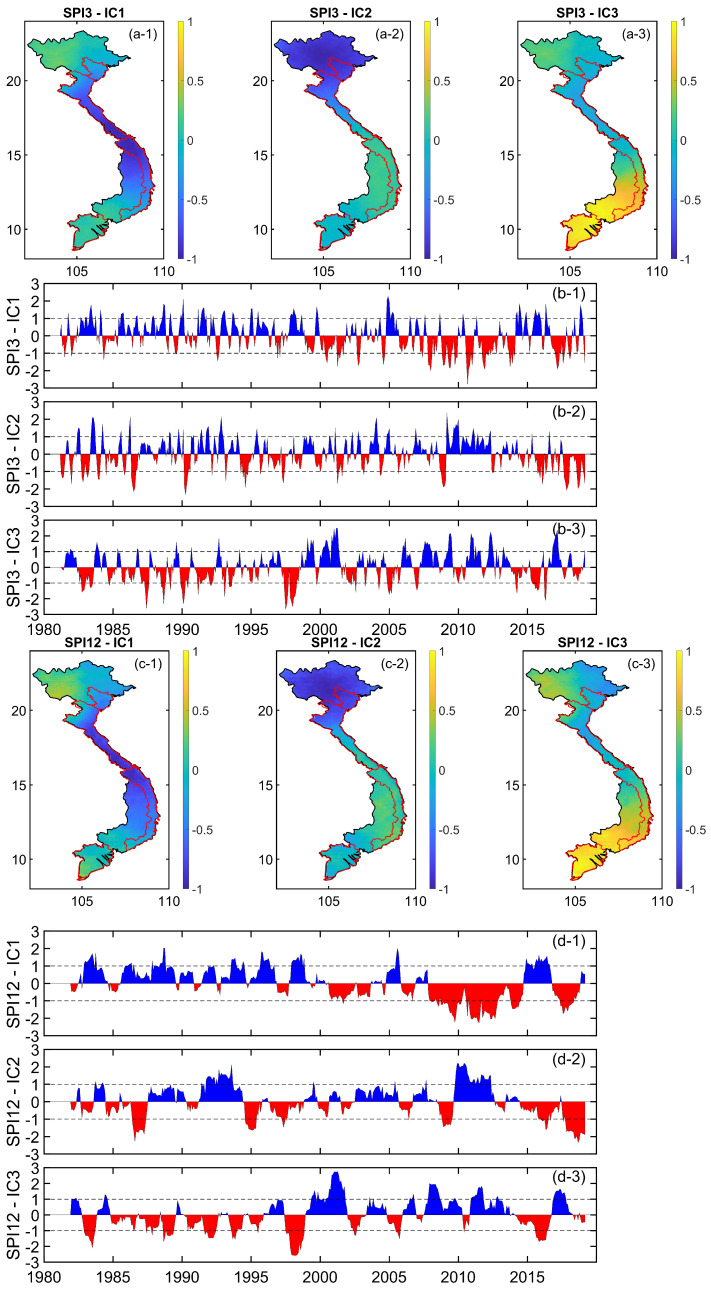
Independent component analysis (ICA) spatial patterns of the first three independent components decomposed from 3-month (**a-1**–**a-3**) and 12-month (**c-1**–**c-3**) time scale CHIRPS SPIs, and their corresponding temporal patterns: SPI3 (**b-1**–**b-3**), SPI12 (**d-1**–**d-3**).

The ICA analysis was then applied to the SPI3 and SPI12 to extract their dominant components, which are shown in Figure 7a,c for the spatial patterns and Figure 7b,d for the temporal patterns (i.e., the time series of the SPI). The first three leading components of the CHIRPS SPI3 and SPI12 after analysis accounted for 99.9% of the total variance. The temporal patterns according to independent components shown in Figure 7b,d indicate the change of the SPI3 and SPI12 in time that is useful for detecting the climate extremes (i.e., floods/droughts) periods, while the spatial patterns shown in Figure 7a,c indicate the correlation between pixels in terms of the magnitude of the SPI3 and SPI12.

In the case of the first independent component (see Figure 7a-1,c-1), it is shown that the magnitude of the SPI3 and SPI12, which is relevant to their maximum variability, was similar between the north and south of Vietnam, which is different from that of the central area. Conversely, in both the second and third dominant independent components (see Figure 7a-2,a-3,c-2,c-3), similar patterns could be seen either in the upper part (latitude larger than ∼15${}^{\circ }$ N) or the lower part (latitude small than ∼15${}^{\circ }$ N), which were in opposite (i.e., positive vs. negative) magnitudes.

### 3.3. The Link between Soil Moisture Variability with Rice Yield

**Figure 8:**
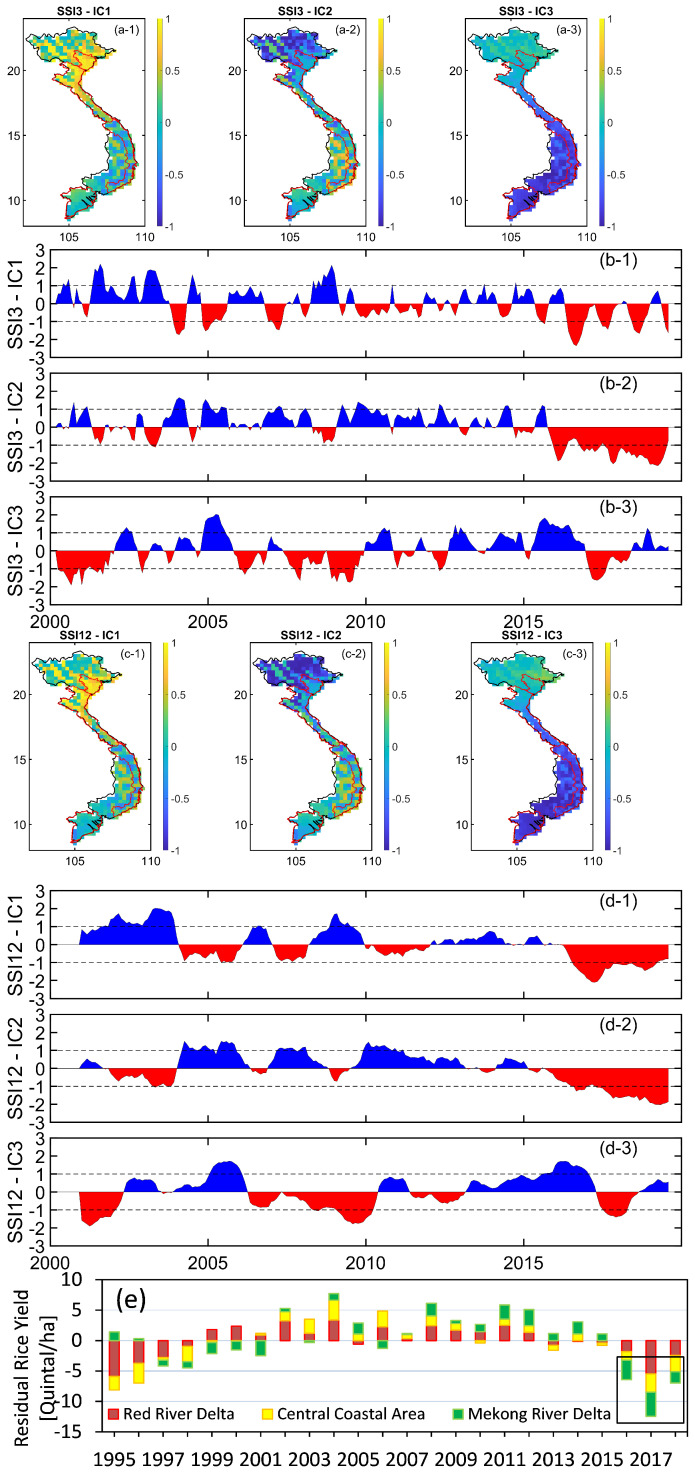
ICA spatial patterns of the first three independent components decomposed from the 3-month (**a-1**–**a-3**) and 12-month (**c-1**–**c-3**) time scale GLDAS SSI, and their corresponding temporal patterns: SSI3 (**b-1**–**b-3**), SSI12 (**d-1**–**d-3**), and the residual rice yield after removing the linear trend over the main rice-producing areas of Vietnam (**e**). The rice yield in (**e**) was derived by dividing the rice production by the planted area.

Precipitation after reaching the Earth’s surface splits into different forms such as surface water, groundwater, soil moisture, canopy wetness, or ice/snow, of which, soil moisture has direct relevance to agricultural productivity. With the aim at the examination of drought effects on rice yield over Vietnam, the 3-month and 12-month time scale GLDAS standardized soil moisture indices (hereinafter SSI3 and SSI12) were computed. The SSI3 and SSI12 were computed for each pixel, then the ICA analysis was applied to the computed SSI3 and SSI12 to decompose the signal into different independent components and retain the dominant ones. The spatial patterns of the first three dominant components of SSI are shown in Figure 8a (SSI3) and Figure 8c (SSI12), with the corresponding temporal patterns shown in Figure 8b (SSI3) and Figure 8d (SSI12). For both indices of SSI3 and SSI12, the first three leading independent components accounted for 99.8% of the total variance.

An agreement in both spatial and temporal patterns of dominant signals was found from those computed from SSI3 and SSI12. With the first dominant signal, the maximum variability in SSI was found in the northern part (latitude larger than ∼15${}^{\circ }$ N), which is likely dominated by the dry period in the northern part between September and January (see Section 3.1). An agreement in the spatial patterns between the second and third dominant signals was found, which indicates distinct patterns between the north (latitude larger than ∼15${}^{\circ }$ N) and the south (latitude smaller than ∼15${}^{\circ }$ N) of the Vietnamese mainland (see Figure 8a-2–a-3,c-2–c-3). These reveal the maximum variability of SSI occurring in the southern part, which likely reveals the dry period during November–April (see Section 3.1).

The temporal patterns of the leading components of SSI shown in Figure 8b (SSI3) and Figure 8d (SSI12) indicate the change of the SSI in time. Consistent trends were found between those computed from the two indices of SSI3 and SSI12, which indicate significant drought in terms of soil moisture during the 2016–2019 period from both of the first two dominant signals. Figure 8e shows the residual rice yield after removing the linear trend over three areas: the RRD, the Central Coast area, and the MRD corresponding to the areas bounded by the red borders in Figure 8a,c. This shows a reduction in the residual rice yield over all three areas during the period of 2016–2018 that has been traced back to weather concerns, e.g., drier than usual climate reported during the 2018–2019 period (https://www.world-grain.com/articles/12759-vietnams-rice-production-estimate-drops-on-weather-concerns, accessed on 1 December 2021). The 2016-2018 reduction in residual rice yield may reflect the long-term periodic change with a similar reduction found between 1995 and 1998.

A link between the reduction of residual rice yield during the period 2016–2018 (highlighted by the black rectangle in Figure 8e) and the first two independent components of SSI was found, which indicates a shortage in both SSI3 (shown in Figure 8b) and SSI12 (shown in Figure 8d) soil moisture during the same time period. Therefore, it can be concluded that the SPI is useful for detecting climate extreme (floods/droughts) periods, while the SSI can be of assistance for investigating the influences of climate extremes on rice productivity/yield over Vietnam.

## 4. Conclusions

In this study, the spatio-temporal variability of remotely-sensed gridded precipitation (CHIRPS) and soil moisture (GLDAS) products was investigated and their indications of drought periods and influences on rice yield over Vietnam during the time period 1981–2019 were assessed. The major findings were:

(i) The spatio-temporal variability of the CHIRPS precipitation confirmed that the rainy season lasts from April/May until September/October, which starts and ends earlier in the northern part (latitude above 20${}^{\circ }$ N) around February and September, respectively, with July–August receiving the highest amount of precipitation over most parts of the country. Areal mean precipitation computed over the four main rice producing areas indicates different peaks of precipitation, in which the months with the highest amount of precipitation were later from north to south.
(ii) An agreement between monthly CHIRPS precipitation and monthly GLDAS soil moisture was found in which the wetter period in terms of soil moisture over the northern and NCC areas was between February and August, while that over the southern and SCC areas started later in May every year. Similarly, the same peaks (i.e., months) of the highest monthly precipitation and soil moisture were found between the four main rice producing areas. However, the spatial patterns of annual mean precipitation and soil moisture disagreed, which can be attributed to different factors other than precipitation affecting the amount of moisture in soil layers, e.g., temperature, irrigation, and drainage systems, which are inconsistent between areas.
(iii) The drought conditions were assessed by the 3-month and 12-month SPI and the impacts of climate extremes on rice yield were assessed via the 3-month and 12-month SSI, both with the ICA analysis. The results showed that the SPI is useful for capturing climate extremes and that SSI is useful in identifying the influences of climate extremes on rice production in Vietnam. During the 2016–2018 period, there existed a reduction in the residual rice yield that was consistent with a decrease in soil moisture, particularly in the first two ICA decomposed leading components.
(iv) Given that precipitation and, to a large extent, soil moisture control plant growth, their increase or decrease due to the impacts of climate extremes are bound to affect all crop growth and production. This study, although specific to rice production, could be undertaken for any crop. The limitation, however, is that access to in situ soil moisture and, to some extent, precipitation products in many countries is challenging. In this scenario, the method was restricted to the use of satellite and/or hydrological model products.

## Figures and Tables

**Table 1:** Summary of data employed in this study.

Category	Dataset	Temporal Resolution	Spatial Resolution	Time Period	References
Vietnam rice statistics		Annually	Areal aggregation	1995–2018	
Precipitation	CHIRPS	Monthly	$0.05^{\circ }\times 0.05^{\circ }$	January 1981–February 2019	[30]
	TRMM	Monthly	$0.25^{\circ }\times 0.25^{\circ }$	January 1998–June 2019	[31,32,33]
Soil moisture	GLDAS	Monthly	$0.25^{\circ }\times 0.25^{\circ }$	January 2000–July 2019	[34,35,36]
	MERRA-2	Monthly	$0.625^{\circ }\times 0.5^{\circ }$	January 1980–September 2019	[37,38]

**Table 2:** Drought classification based on the SPI.

SPI	Drought Category	Probability %
≥2	Extremely wet	2.3
1.50 to 1.99	Severely wet	4.4
1.00 to 1.49	Moderately wet	9.2
0 to 0.99	Mildly wet	34.1
0 to −0.99	Mild drought	34.1
−1.00 to −1.49	Moderate drought	9.2
−1.50 to −1.99	Severe drought	4.4
≤−2	Extreme drought	2.3

**Figure A1:**
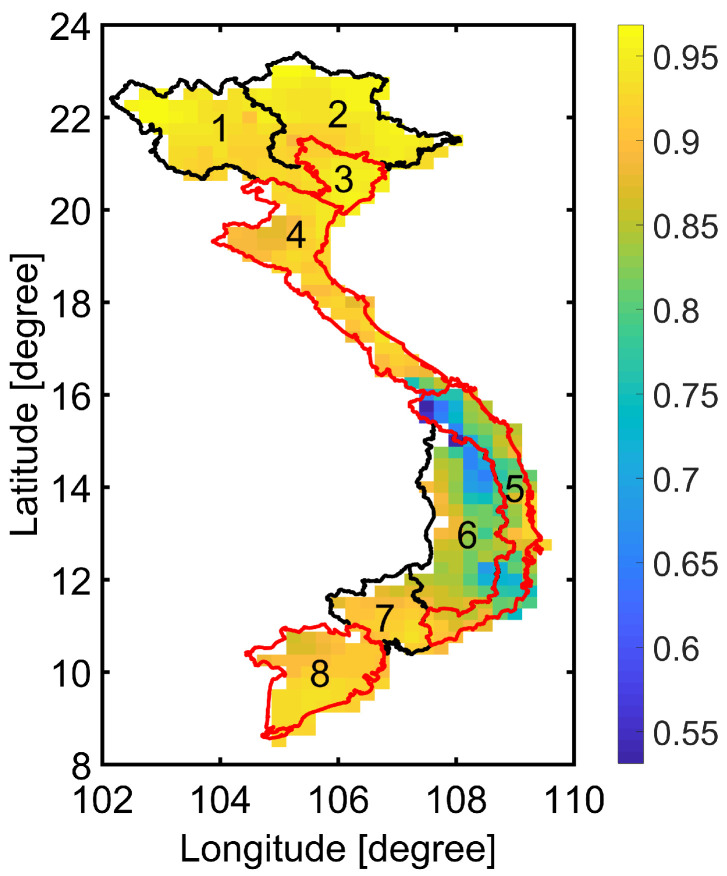
Pixel correlation coefficients between CHIRPS and TRMM precipitation products. The results were computed pixel-by-pixel over the entire Vietnamese territory. The sub-area borders and their order numbers were the same as those in Figure 1. Red borders are associated with the main rice-producing areas investigated in this study.

**Figure A2:**
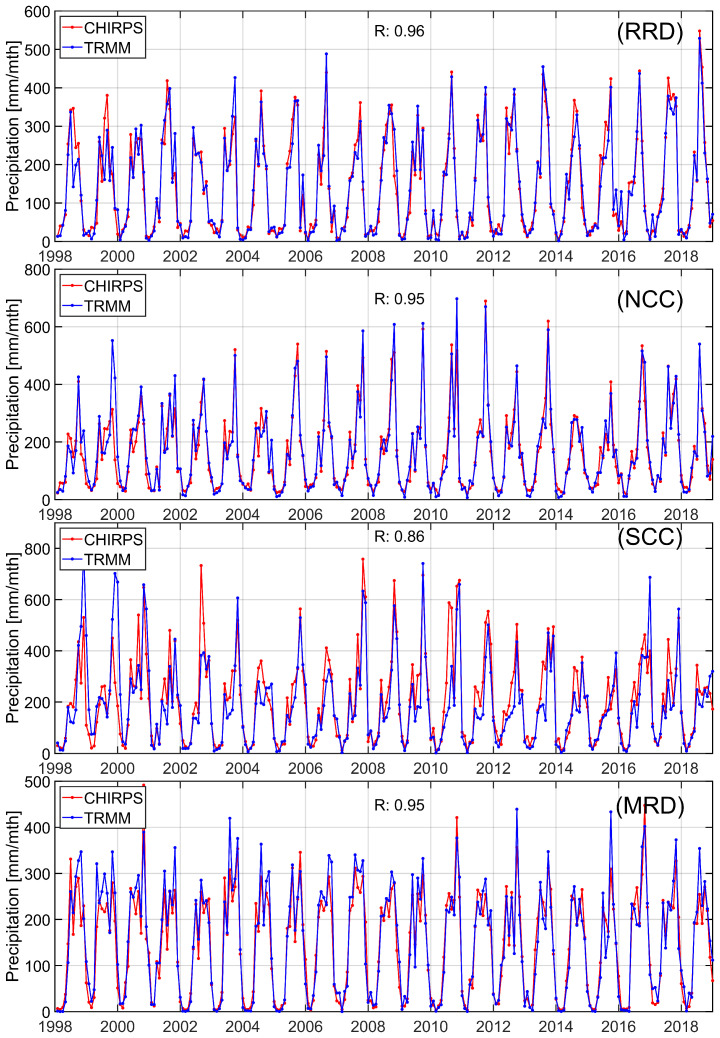
The difference between the areal mean of monthly CHIRPS and TRMM precipitation products averaged over the four main rice-producing areas: the RRD (**top-left**), the NCC area (**top-right**), the SCC area (**bottom-left**), and the MRD (**bottom-right**).

**Figure A3:**
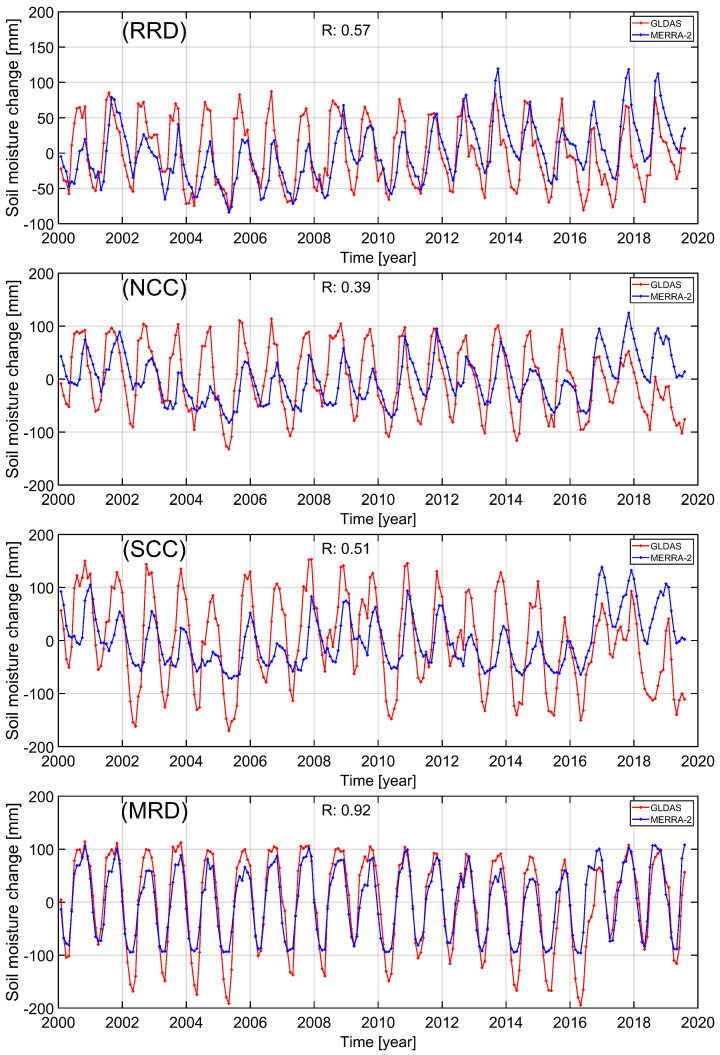
Difference between the areal mean of GLDAS and MERRA-2 soil moisture averaged over the four main rice-producing areas: the RRD (**top-left**), the NCC (**top-right**), the SCC (**bottom-left**), and the MRD (**bottom-right**).

**Figure A4:**
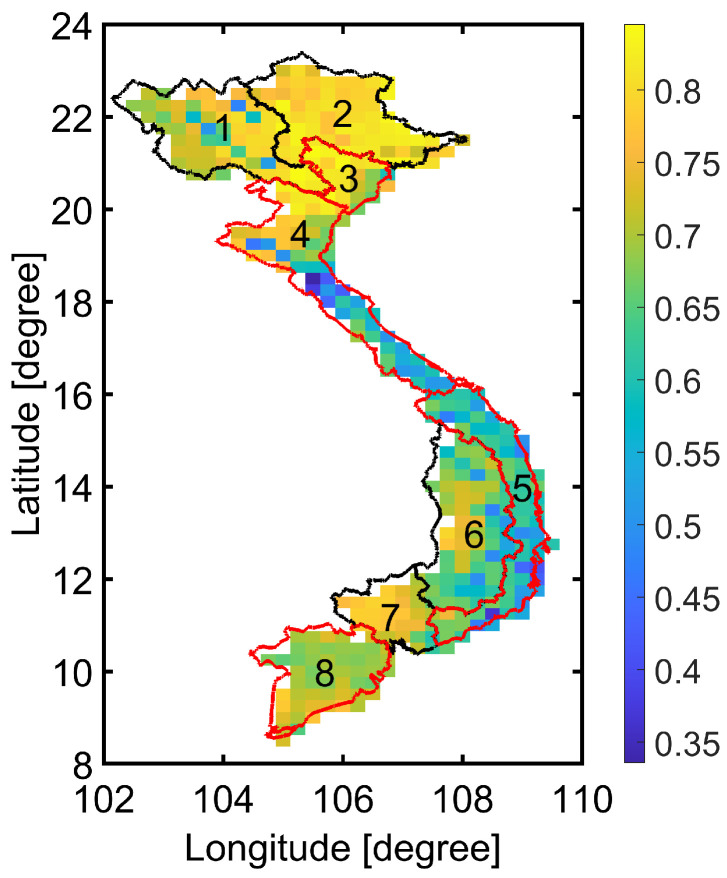
Pixel correlation coefficients between GLDAS soil moisture and TRMM precipitation over the Vietnamese mainland. The sub-area borders and their order numbers are the same as those in Figure 1. Red borders are associated with the main rice-producing areas investigated in this study.

**Figure A5:**
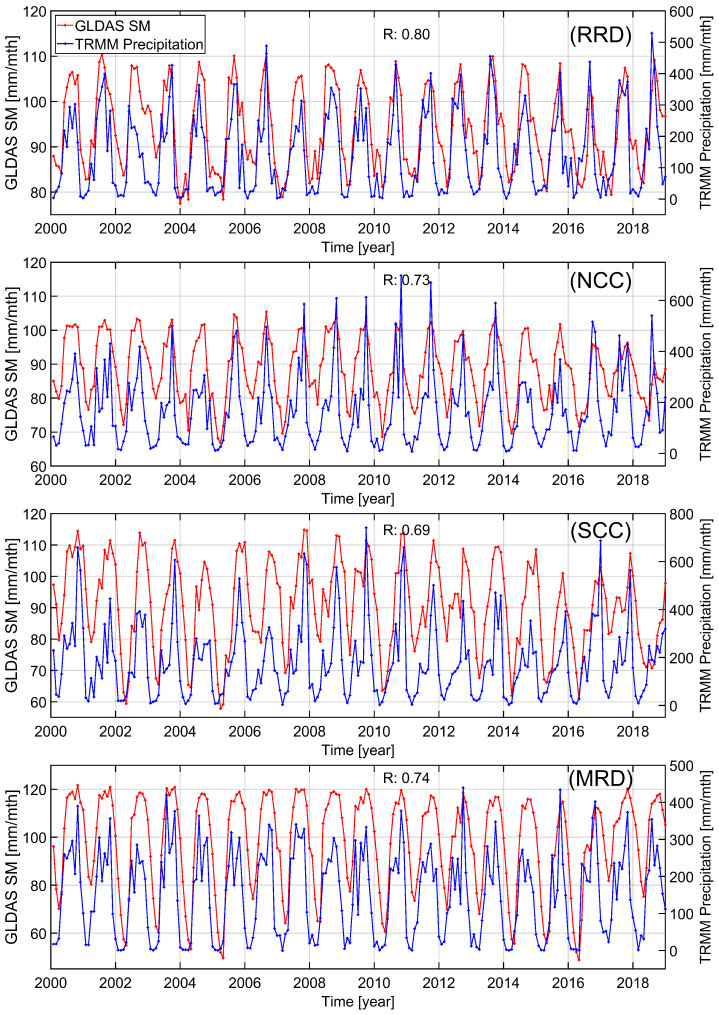
Temporal variability of areal mean GLDAS soil moisture and TRMM precipitation showing seasonal patterns over the four areas of interest: (**top-left**) the RRD, (**top-right**) NCC, (**bottom-left**) SCC, and (**bottom-right**) MRD.

## Data Availability

Data sharing not applicable.

## Appendix A. Consistency between CHIRPS and TRMM Precipitations

CHIRPS and/or TRMM precipitations have been applied in several studies over Vietnam on local-scale areas [14,16,79,80]. In Simon et al. [79], TRMM and CHIRPS precipitation data were verified by ground observations in the Red River Basin in which the upstream part is located in southern China and the downstream part belongs to northern Vietnam. The correlation between CHIRPS, TRMM, and rain gauge data from [79] showed high correlation coefficients of 0.85 and 0.88, respectively, though these values were based on a limited study area. Here, CHIRPS and TRMM precipitation data were inter-compared to examine their consistency over the entire Vietnam, which was then separated into four investigation areas, including RRD, NCC, SCC, and the Vietnamese MRD (see Figure 1 for the regions). These four areas were investigated as they are directly relevant to three main rice-producing areas and are based on their geographical locations (see Figure 1).

The two datasets are truncated to the common 21-year period of January 1998–December 2018 (see Figure 3). Additionally, due to the different spatial resolutions (see Table 1), the CHIRPS dataset is down-sampled to $0.25^{\circ }\times 0.25^{\circ }$ by averaging over 5×5 adjacent pixels with no loss of information as both datasets are provided as a representative of their areal-averaged precipitation within the pixel. Following these two steps, the two datasets are of the same spatial resolution and cover the same time period. The correlation between the two precipitation datasets was first investigated on a pixel-by-pixel basis of which the results are shown in Figure A1. The investigation demonstrates a high consistency with the maximum and minimum correlation coefficients being ∼0.97 and ∼0.53 at a 95% confidence level, respectively, with the lower correlations found in pixels located in the SCC and the Central Highlands areas (see Figure A1).

Due to the fact that this study focuses on the effects of climate variability/change and climate extremes on rice production, a similar investigation was conducted over four areas of RRD, NCC, SCC, and MRD by the areal mean values. To this end, precipitation data were first spatially averaged over each area, then the averaged CHIRPS and TRMM time series were tested for correlation via graphical demonstration and the correlation coefficients. Figure A2 shows the graphical demonstration where the smallest correlation coefficient of 0.86 was found in the SCC area, while the remaining areas exhibited an almost identical value of ∼0.95. This high level of correlation indicates that within these rice growing areas, either of these two products could be useful for analysis of rice production in Vietnam.

## Appendix B. Consistency between GLDAS and MERRA-2 Soil Moisture Products

Due to the lack of in situ data, two soil moisture (SM) products of Noah GLDAS 2.1 and MERRA-2 were cross-compared for their consistency. A test of pixel correlation between the two products could not be made due to their disagreement in spatial resolution (see Table 1). Instead, they were tested for the areal mean time series comparison over four main rice-producing areas, i.e., the RRD, NCC, SCC, and MRD (Figure 1). Here, we tested the consistency in the root zone soil layer (up to 100 cm) because it is the only variable in both products (see Section 2.2.3). However, MERRA-2 SM is provided to a 0.75 m depth only in some areas of the globe. The comparison between absolute SM of the two datasets may lead to unreasonable results. Additionally, with the aim of assessing the influence of climate change, the SM change was adopted in this test. For each area, SM change was derived for the two products by subtracting mean values over the time span prior to computing the spatial average. The time series plots of SM changes are depicted in Figure A3 (right panel), which demonstrate an agreement in the seasonal patterns. The results indicate higher correlations in the MRD area than in the other areas of RRD, NCC, and SCC.

## Appendix C. Correlation between Precipitation and Soil Moisture Products

After reaching the Earth’s surface, precipitation splits into different forms, e.g., surface water, groundwater, or soil moisture. Therefore, precipitation and soil moisture have different correlations between areas. Here, the correlation between precipitation and soil moisture products was tested over the Vietnamese territory. For this test, GLDAS was adopted as a representative of soil moisture due to its higher spatial resolution ($0.25^{\circ }$) than that of MERRA-2 ($0.625^{\circ }\times 0.5^{\circ }$). In Appendix A, CHIRPS and TRMM were demonstrated to be highly consistent, particularly over the four areas of interest, i.e., the RRD, NCC, SCC, and MRD. For this reason, TRMM was utilized in this investigation as a representative of precipitation due to its equivalent spatial resolution to that of GLDAS soil moisture (see Table 1).

Because of the difference in time spans, the two datasets, i.e., TRMM precipitation and GLDAS soil moisture, were truncated to be bounded within the common January 2000–December 2018 time period. With a particular interest in agricultural application (rice yield), the GLDAS soil moisture of the 10–40 cm soil layer was exploited. The correlation coefficients were then calculated for all pixels limited within the Vietnamese territory of which the spatial patterns are shown in Figure A4. The results show correlation coefficients at different magnitudes with the maximum and minimum values being ∼0.85 and ∼0.34 at a 95% confidence level, respectively, and lower values were found in the Northwest (1), Central Coast (4, 5), and Central Highlands (6) areas. Areas exhibiting lower correlation coefficients occupied either the mountainous areas (1, 6) or the coastal areas (4, 5), see Figure 1. This is probably because high relief characteristics in mountainous areas reduce the ability of soil to store water or water sources other than precipitation, e.g., seawater, is contributed in coastal areas, both of which result in lower correlation between precipitation and soil moisture.

With the aim of focusing on the main rice-producing areas, the time series of TRMM precipitation and GLDAS soil moisture were spatially averaged over the four areas of RRD, NCC, SCC, and MRD. These areal mean time series were subsequently tested for their correlation via the time variability and the correlation coefficients (*R*) shown in Figure A5. An agreement in seasonal patterns was found between the time variability of areal mean TRMM precipitation and GLDAS soil moisture in all four areas, with more stable variability over years found in the deltas (top-left and bottom-right) than the Central Coast areas (top-right and bottom-left). The correlation coefficients varied between 0.69 and 0.80 with the deltas (RRD, MRD) exhibiting higher correlations than the coastal areas (NCC, SCC).
